# Identification and Profiling of microRNAs and Their Target Genes from Developing Caprine Skeletal Muscle

**DOI:** 10.1371/journal.pone.0096857

**Published:** 2014-05-12

**Authors:** Yanhong Wang, Chunlei Zhang, Xingtang Fang, Yulong Zhao, Xiaohui Chen, Jiajie Sun, Yang Zhou, Jianjin Wang, Yongan Wang, Xianyong Lan, Hong Chen

**Affiliations:** 1 College of Animal Science and Technology, Northwest A&F University, Shaanxi Key Laboratory of Molecular Biology for Agriculture, Yangling, Shaanxi, China; 2 Institute of Cellular and Molecular Biology, Jiangsu Normal University, Xuzhou, Jiangsu, China; Chinese Academy of Fishery Sciences, China

## Abstract

Goat is an important agricultural animal for meat production. Functional studies have demonstrated that microRNAs (miRNAs) regulate gene expression at the post-transcriptional level and play an important role in various biological processes. Although studies on miRNAs expression profiles have been performed in various animals, relatively limited information about goat muscle miRNAs has been reported. To investigate the miRNAs involved in regulating different periods of skeletal muscle development, we herein performed a comprehensive research for expression profiles of caprine miRNAs during two developmental stages of skeletal muscles: fetal stage and six month-old stage. As a result, 15,627,457 and 15,593,721 clean reads were obtained from the fetal goat library (FC) and the six month old goat library (SMC), respectively. 464 known miRNAs and 83 novel miRNA candidates were identified. Furthermore, by comparing the miRNA profile, 336 differentially expressed miRNAs were identified and then the potential targets of the differentially expressed miRNAs were predicted. To understand the regulatory network of miRNAs during muscle development, the mRNA expression profiles for the two development stages were characterized and 7322 differentially expressed genes (DEGs) were identified. Then the potential targets of miRNAs were compared to the DEGs, the intersection of the two gene sets were screened out and called differentially expressed targets (DE-targets), which were involved in 231 pathways. Ten of the 231 pathways that have smallest *P*-value were shown as network figures. Based on the analysis of pathways and networks, we found that miR-424-5p and miR-29a might have important regulatory effect on muscle development, which needed to be further studied. This study provided the first global view of the miRNAs in caprine muscle tissues. Our results help elucidation of complex regulatory networks between miRNAs and mRNAs and for the study of muscle development.

## Introduction

MicroRNAs (miRNAs) are small (∼22 nucleotides) noncoding RNAs which are highly conserved among mammals [1]. They bind primarily to the 3′-UTR of target mRNAs to regulate their translation and accelerate their decay [1], [2]. Hundreds of miRNAs have been discovered in recent years and several have been functionally characterized. miRNAs play critical fine-tuning roles in diverse biological processes, such as cell proliferation and differentiation [3], [4], tumorigenesis [5], the morphogenesis of special organs [6] and viral defense [7].

Skeletal muscle occupies approximately 40% of the body weight [8], and its development is a complex process involving multiple factors which regulate the proliferation of myoblasts, their exit from the cell cycle and subsequent differentiation into multinucleated myotubes [9]. The discovery of miRNAs has open up a new field of factors controlling skeletal muscle development. Previous studies showed that miRNAs had important effect on skeletal muscle development. They could regulate myogenesis or hypertrophy under physiological and patho-physiological conditions. Skeletal muscle related miRNAs influence multiple facets of muscle development and function through the regulation on key genes controlling myogenesis [10]–[12]. Recently, several miRNAs that highly-enriched in skeletal muscle have been identified. The muscle specific miR-1, miR-133 and miR-206 are perhaps the most studied and best-characterized miRNAs. They play important roles in myoblast proliferation, differentiation, and hypertrophy [10]–[11], [13]–[14]. In addition to muscle specific miRNAs, non-muscle-specific miRNAs also regulate skeletal muscle differentiation in multiple ways. Muscle differentiation-related genes such as MyoD, MEF2, Pax3 and YY1, are regulated by these non-muscle-specific miRNAs [15]–[18]. For example, miR-699a is able to inhibit skeletal muscle differentiation,and shows down regulated expression during skeletal muscle differentiation [15]. Thus, globally identifying and characterizing skeletal muscle miRNAs during various phases of muscle development will significantly enhance our understanding of skeletal muscle biology and function.

Previous studies indicated that many miRNAs have multiple target genes and most of the genes are targeted by multiple miRNAs [19]. For example, YY1 is targeted by miRNA-1 [20] and miRNA-29a [21]. YY1 can inhibit skeletal muscle cell differentiation by inhibiting the synthesis of late-stage differentiation genes including muscle creatine kinase and myosin heavy chain [22]. Pax3 was reported to be targeted by miRNA-1 [11], miRNA-27b [23] and miRNA-206 [24]. High levels of Pax3 interfere with muscle cell differentiation, in both the embryo and adult [23]. Skeletal muscle stem cells are regulated by Pax3/7. Pax7 was targeted by miRNA-1 [25], miRNA-206 [26] and miRNA-486 [26]. On the other hand, the experimentally proved target genes for miRNA-1 include YY1, HDAC4, Cx43, Pax3 and Pax7. Thus, muscle miRNAs and their target genes may integrate into complex regulatory networks, an integrated analysis of expression of miRNAs and their targets can be helpful to identify miRNA/mRNA modules which may be involved in muscle development regulation.

Goat is one of the most important meat-producing livestock animals grown worldwide. Many studies have focused on miRNA, and a suite of miRNAs highly-enriched in skeletal muscle has been identified [14], [27]–[30]. However, few works have been done on the identification of goat muscle miRNAs. Therefore, global identification of the known and novel miRNAs involved in goat skeletal muscle development is essential. In addition, the mRNA profiles were analyzed in this study. Based on these, we briefly analyzed the regulation network of miRNA-mRNA, which will significantly enhance our understanding the effect of miRNAs on muscle development for goat.

## Materials and Methods

### Ethics statement

Huanghuai Goats in present study were bought from HeQiao caprine Co., Ltd. Huanghuai goat is famous for its meat-producing in China. All animals were raised and fed under the same standard management conditions according to the No. 5 proclamation of the Ministry of Agriculture, P. R. China. Sample collection was approved by the Animal Care Commission of the College of Animal Science and Technology, Northwest A&F University.

### Tissue collection and high-throughput sequencing

Ninety day embryos were collected from a flock of ewes, which were estrus synchronized, at 90 days of pregnancy (gestation period 150 days). Embryos were collected into sterile physiological saline immediately after removal from the reproductive tract of slaughtered ewes at an abattoir. Longissimus thoracis muscle tissues were collected from fetal and six month old Huanghuai goat, and immediately snap-frozen in liquid nitrogen, then stored at −80°C until use.

Total RNAs were extracted from 8 fetal and 8 six month old Huanghuai goat longissimus thoracis. The total RNA was isolated from each sample using the Trizol reagent (Takara, Dalian, China). RNA was quantified using the Nano-Drop ND-2000 spectrophotometer (NanoDrop products, Wilmington, USA) and checked for purity and integrity in a Bioanalyzer-2100 (Agilent Technologies, Inc., Sant a Clara CA, USA). Then the RNAs from fetal and six month old goats were pooled, respectively. Subsequently, low molecular weight RNAs were separated by 15% polyacrylamide gel electrophoresis (PAGE), and RNA molecules in the range of 18–30 nt were enriched and ligated with proprietary adapters to the 5′and 3′termini. A reverse transcription reaction followed by low cycle PCR was performed to obtain sufficient product for Solexa technology (Beijing Genomics Institute, China). In the present study, two miRNA libraries were constructed.

### Small RNA sequence analysis

After getting rid of reads with 5' primer contaminants and without 3' primer, removal of redundancy and reads smaller than 18 nt, the clean reads were mapped to the latest caprine genome assembly [http://goat.kiz.ac.cn/GGD/download.htm] using the program SOAP [31]. In order to determine the compositions of small RNA sample, the length distribution analysis was conducted using caprine mRNA [http://goat.kiz.ac.cn/GGD/download.htm], and CDS [http://goat.kiz.ac.cn/GGD/download3.htm], RepeatMasker [http://www.re peatmasker.org] and Sanger Rfam data (version 10.1), according to the length of the small RNA. For example, miRNA is normally 21 nt or 22 nt, siRNA is 24 nt. Subsequently, the remaining reads were searched against the Sanger miRBase (version 19.0) to identify the known miRNAs. Only those small RNAs whose mature and precursor sequences perfectly matched known bovine miRNAs and ovine miRNAs in miRBase were considered to be known caprine miRNAs. To discover potential novel miRNA precursor sequences, unique sequences that have more than 10 hits to the genome or match to known non-coding RNAs were removed. Then the flanking sequences (150 nt upstream and downstream) of each unique sequence were extracted for secondary structure analysis with M fold [http://www.bioinfo.rpi.edu/applications/mfold] and then evaluated by Mireap [http://source forge.net/project s/mireap/]. After prediction, the resulting potential miRNA loci were examined carefully based on the distribution and numbers of small RNAs on the entire precursor regions. Those sequences residing in the stem region of the stem-loop structure and ranging between 16–30 nt with free energy hybridization lower than -18 kcal/mol were considered. Then, all novel miRNA candidates were further subjected to MiPred (http://www.bioinf.seu.edu.cn/miRNA/) to filter out pseudo-pre-miRNAs [32].

### MicroRNA expression analysis

To find out the differentially expressed miRNAs, we compared the known and novel miRNA expression profile between two samples. The expression of miRNA was shown in two samples by plotting a Log_2_-ratio figure and a Scatter Plot. The procedures were shown as below: (1) Normalize the expression of miRNA in two samples (fetal and six month longissimus thoracis) to get the expression of transcript permillion. Normalized expression (NE)  =  actual miRNA count/total count of clean reads. (2) Calculate fold-change and *P*-value from the normalized expression. Then generate the Log_2_-ratio plot and scatter plot. Fold-change formula: fold change  =  log_2_ (fetal NE/six month NE). *P*-value formula:
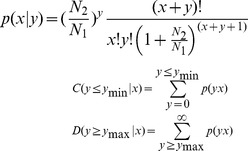



The x and y represented normalized expression levels, and the N_1_ and N_2_ represented total count of clean reads of a given miRNA in small RNA libraries of the fetal and six month stage, respectively.

### Using Stem-loop RT-qPCR method to validation of differentially-expressed miRNAs

In order to evaluate the repeatability and reproducibility of miRNA expression data obtained by Small RNA-Seq, a Stem-loop real-time reverse transcription polymerase chain reaction (RT-PCR) with SYBR Green was used for the analysis of miRNA expression [33]. The isolated RNA of individual samples was converted to cDNA with a RT primer mixture (250 nM) using PrimeScript RT reagent Kit with gDNA Eraser (TaKaRa, Dalian, China) in a total volume of 20µl containing 1µg of total RNA. The cDNA was then used for real-time PCR quantification of miRNA using the miRNA specific forward primer and the universal reverse primer. 18 S rRNA was used as the reference gene in the qRT-PCR detection of goat miRNAs. Real-time quantitative PCR was performed using an ABI Stepone plus Real Time Detection System (Applied Biosystems, Inc., Foster City, CA) and SYBR Green PCR Master Mix (TaKaRa, Dalian, China) in a 20µl reaction volume. Each sample was analyzed by triplicate. The PCR mix included 100 ng cDNA for each miRNA, 0.4µM forward and reverse primers, and 10µl 2× SYBR Green PCR Master Mix, thermal cycle was: 95°C for 30 s, followed by 40 cycles of 95°C for 5 s, 60°C for 30 s. The threshold cycle (Ct) was defined as the cycle number at which the fluorescence intensity passed a predetermined threshold. The quantification of each miRNA relative to 18 S rRNA gene was calculated.

### RNA-Seq (Transcriptome)

The mRNA was isolated from total RNA (described as above) to construct cDNA libraries. The cDNA was synthesized using the mRNA fragments as templates. Agilent 2100 Bioanaylzer and ABI Stepone Plus Real-Time PCR System were used in quantification and qualification of the sample library. The library was sequenced using Illumina HiSeq TM 2000. Then, the primary sequencing data was subjected to quality control (QC). After remove reads with adapters, reads in which unknown bases are more than 5% and low quality reads (we define the low quality base to be the base whose sequencing quality is no more than 10), raw reads are filtered into clean reads, which were aligned to the latest goat genome assembly [http://www.ncbi.nlm.nih.gov/bioproject/PRJNA158393] using SOAP aligner/SOAP2 [34]. No more than 5 mismatches are allowed in the alignment. Mapping was performed to the entire genome and gene.

### Screening of DEGs and validated by RT-qPCR

Referring to Audic and Claverie (1997) [35], we have developed a strict algorithm to identify differentially expressed genes between two samples, and the method used is described as follow: denote the number of unambiguous clean tags (which means reads in RNA_Seq) from gene A as x, given every gene's expression occupies only a small part of the library, x yields to the Poisson distribution:
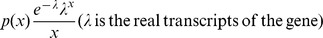



The total clean tag number of the FC is N1, and total clean tag number of SMC is N2; gene A holds x tags in FC and y tags in SMC. The probability of gene A expressed equally between two samples can be calculated with: 
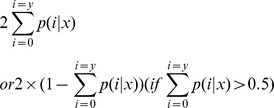


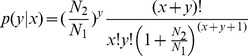



*P*-value corresponds to differential gene expression test. Since DEG analysis generate a large multiplicity problems in which thousands of hypothesis (is gene x differentially expressed between the two groups) are tested simultaneously, correction for false positive (type I errors) and false negative (type II) errors are performed using [36] FDR method. This is a standard approach for multiple hypothesis correction for most of the common used software for differential expression analysis. We used *‘FDR≤0.001 and the absolute value of Log_2-_Ratio≥1’* as the threshold to judge the significance of gene expression difference.

To validate the sequence data, the isolated RNA of individual samples was converted to cDNA with a RT primer mixture (250 nM) using Prime Script RT reagent Kit with gDNA Eraser (TaKaRa, Dalian, China) in a total volume of 20µl containing 1µg of total RNA. The cDNA was then used for real-time PCR quantification of candidate DEGs. GAPDH was used as the reference gene in the qRT-PCR detection.

### microRNA-mRNA integrated analysis

After small RNA sequencing and RNA-Seq (Transcriptome) analysis of the paired samples, the integrated analysis of differentially-expressed miRNAs and their predicted target genes were performed. Firstly, we predicted the potential targets of known miRNAs. The putative target genes for known miRNAs were searched by aligning the miRNA sequences with 3′UTR sequences of goat mRNA sequence. The target prediction tool RNAhybrid (http://bibiserv.techfak.uni-bielefeld.de/rnahybrid) [37] was employed and the rules used for target prediction are based on those suggested by Allen et al. (2005) and Schwab et al. (2005) [38], [39], details were in accordance with Ji et al. (2012) [40]. Secondly, the target genes of differentially expressed miRNAs were sorted out. Then we compared these target genes with DEGs, and sorted out the intersections of the two gene sets, called them DE targets. Then we analyzed the regulating networks between differentially-expressed miRNAs and DE targets.

### Pathway analysis of differential expression targets

To comprehensively describe the properties of the integrated analysis results, database for Annotation, Visualization, and Integrated Discovery (DAVID) [41]–[42] was used to perform pathway enrichment analysis of DE targets. The results of this integrated analysis of different functional databases (e.g. GO, KEGG pathways, SP-PIR keywords). FDR was used to correct the *P*-value. KEGG analysis has satisfaction values of *P*<0.05 and FDR≤0.05. Genes with FDR≤0.05 are considered as significantly enriched target gene candidates.

## Results

### Small RNA libraries from goat longissimus thoracis

**Figure 1:**
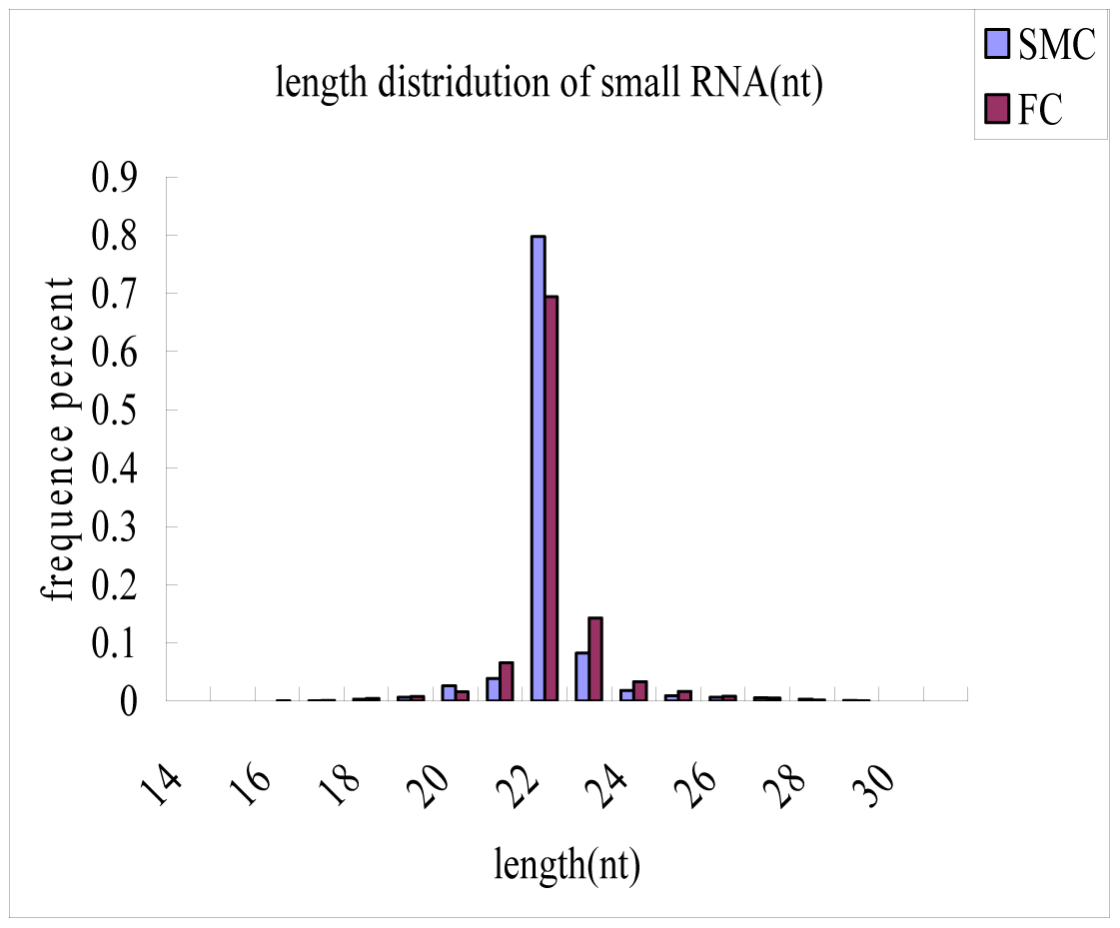
Length distribution of small RNAs in SMC and FC libraries. SMC: six month old caprine muscle tissue; FC: fetal caprine muscle tissue.

To identify the small RNAs involved in goat muscle development, total RNAs from goat longissimus thoracis at fetal and six month-old stages were used to construct small RNA libraries for deep sequencing. After deleting some contaminant reads, we obtained 15,627,457 clean reads from the fetal caprine (FC) library and 15,593,721 clean reads from the six month old caprine (SMC) library (Table 1). Length distribution analysis showed that most reads ranged from 21 to 23 nt. The percentage of the 22 nt reads in the total reads accounted for 69.42% of the FC library and 79.75% of the SMC library (Figure 1)**.** All Solexa reads were aligned against caprine genome assembly using the program SOAP, thesoap-v0-r2-M0-aclean.fa-Dref_genome.fa.index-omatch_genome.soap. miRNA is the most abundant RNA species in both libraries and represents 81.52% and 82.94 reads in the FC library and the SMC library (Table 2), respectively.

**Table 1:** Summary of small RNA sequencing date.

Type	Fetal caprine muscle tissue		Six month-old caprine muscle tissue	
	Count	%	Count	%
total_reads	15731062		15676284	
high_quality	15685279	100%	15630959	100%
3'adapter_null	2683	0.02%	3257	0.02%
insert_null	2119	0.01%	636	0.00%
5'adapter_contaminants	19517	0.12%	17252	0.11%
smaller_than_18 nt	33482	0.21%	16062	0.10%
polyA	21	0.00%	31	0.00%
clean_reads	15627457	99.63%	15593721	99.76%

**Table 2:** Distribution of the genome-mapped sequence reads in small RNA libraries.

Locus	Fetal caprine muscle tissue		Six month-old caprine muscle tissue	
	Unique sequences	Reads	Unique sequences	Reads
Total	317200 (100%)	15627457 (100%)	249940(100%)	15593721 (100%)
miRNA	4229 (1.33%)	12740265 (81.52%)	2679 (1.07%)	12934004 (82.94%)
exon_antisense	892 (0.28%)	965 (0.01%)	396 (0.16%)	430 (0.00%)
exon_sense	28465 (8.97%)	29875 (0.19%)	35305 (14.13%)	38078 (0.24%)
intron_antisense	2087 (0.66%)	5792 (0.04%)	1045 (0.42%)	2431 (0.02%)
intron_sense	12410 (3.91%)	54645 (0.35%)	6261 (2.51%)	34197 (0.22%)
rRNA	42174 (13.30%)	435237 (2.79%)	38504 (15.41%)	355165 (2.28%)
scRNA	15 (0.00%)	17 (0.00%)	10 (0.00%)	10 (0.00%)
snRNA	2057 (0.65%)	6055 (0.04%)	1542 (0.62%)	4218 (0.03%)
snoRNA	1649 (0.52%)	7294 (0.05%)	1001 (0.40%)	4476 (0.03%)
tRNA	8800 (2.77%)	54890 (0.35%)	8510 (3.40%)	65835 (0.42%)
unann	214422 (67.60%)	2292422 (14.67%)	154687 (61.89%)	2154877 (13.82%)

### Identification and profiling of known caprine miRNAs

Because caprine miRNAs database is not available in *miRbase version 19.0* (http://www.mirbase.org/index.shtml), we compared tentative caprine miRNA sequences with the known bovine and ovine sequences. There are currently 821 miRNA precursors and 858 mature miRNAs containing 605 miRNAs, 127 miRNA-5p, 126 miRNA-3p in currently miRBbase. By Blastn searched against the miRBbase19.0, 4,283 unique sequences (12,740,411 reads) were annotated as miRNA candidates in the FC library as well as 2720 unique sequences (12,934,111 reads) in the SMC library, the rest were unannotated. The caprine miRNA candidates were then clustered into 457 and 384 categories corresponding to 437 and 383 independent genomic loci in the two libraries (Table 3 **)**.

**Table 3:** Summary of known miRNAs in each sample.

	miR	miR-5p	miR-3p	pre-miRs	Unique matched to pre-miRs	Read matched to pre-miRs
Known miRs	605	127	126	821		
Fetal caprine muscle tissue	291	82	84	437	4283	12740411
Six month caprine muscle tissue	245	64	75	383	2720	12934111

**Figure 2:**
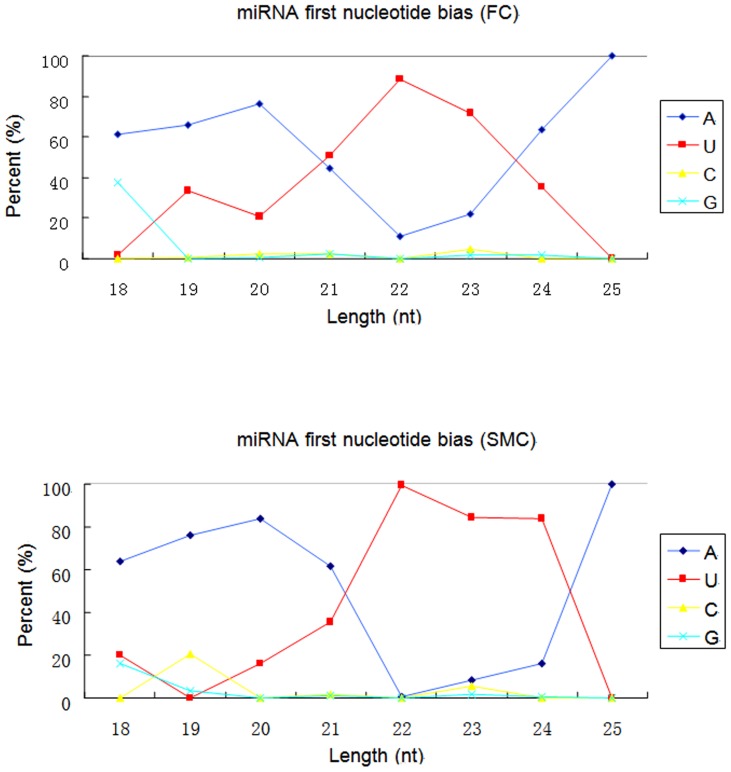
Nucleotide bias of the first base in different sequence lengths. First nucleotide bias in different sequence lengths from 18–25 nt for known miRNAs; SMC: six month-old caprine muscle tissue; FC: fetal caprine muscle tissue.

**Figure 3:**
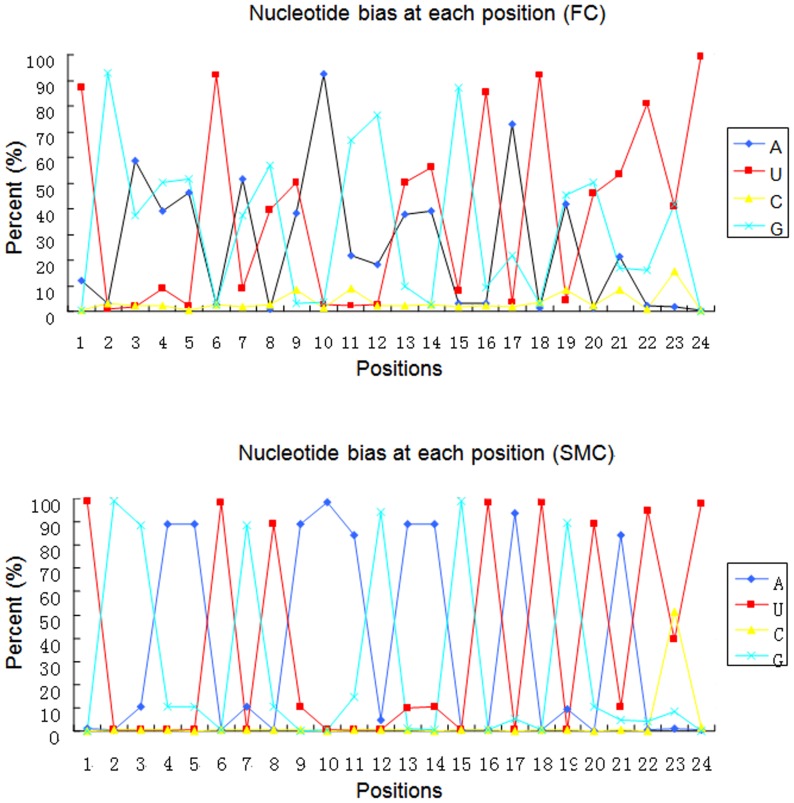
Nucleotide bias at each position. Nucleotide bias at each position (1–24) for known miRNAs. SMC: six month-old caprine muscle tissue; FC: fetal caprine muscle tissue.

We identified 464 known miRNAs in the two libraries (Table S1). 364 known miRNAs are found in both FC and SMC libraries, and the 20 most abundant miRNAs of them were listed in Table 4. 103 miRNAs are displayed in the lowest sequencing frequencies, with no more than 10 reads in FC or SMC library (Table S1). To better characterize the identified known miRNAs, we conducted nucleotide bias analysis. Nucleotide bias shows that first nucleotide bias was different with various sequence lengths in the two libraries. As shown in Figure 2, U was the most frequent first nucleotide in the miRNAs in both the two library at a proportion of 86.88% in FC and 99.0% in SMC. The (A+U) content was found most abundantly, with an average percentage of 99.9% in SMC and 99.29% in FC, while C or G was seldom used as the first nucleotide (Figure 2 and Table S2a). In addition, an analysis of the four nucleotides at each position along the length revealed that there are different nucleotide biases at each position (Figure 3 and Table S2b). For example, U was predominated at positions 1^th^, 6^th^, 16^th^, 18^th^, 22^th^ and 24^th^ in both the two library. The G base had a high frequency in the 2^th^, 3^th^, 7^th^, 12^th^, 15^th^, 19^th^ positions in SMC library. Notable skewing in the distribution of nucleotides at specific positions included significant low G+C content at position 1 (0.71% in FC, 0.09% in SMC) and position 9 (11.63%in FC, 11.58% in SMC).

**Table 4:** Twenty most abundance co-expressed miRNAs.

miR_Name	miRNA abundance		Length	miRNA sequence
	Total reads-FC	Total reads-SMC		
chi-miR-206	3477840	1172192	22	AATGTAAGGAAGTGTGTGGTTT
chi-miR-1	1929430	9986601	22	TGGAATGTAAAGAAGTATGTAT
chi-let-7f	1529359	521706	22	TGAGGTAGTAGATTGTATAGTT
chi-let-7a-5p	1521553	413551	22	TGAGGTAGTAGGTTGTATAGTT
chi-let-7b	821526	225830	22	TGAGGTAGTAGGTTGTGTGGTT
chi-let-7c	632168	93292	22	TGAGGTAGTAGGTTGTATGGTT
chi-miR-199a-3p	180808	24392	22	ACAGTAGTCTGCACATTGGTTA
chi-miR-432	177423	1786	23	TCTTGGAGTAGGTCATTGGGTGG
chi-let-7e	173110	9774	22	TGAGGTAGGAGGTTGTATAGTT
chi-miR-3958-3p	671096	22323	22	AGATATTGCACGGTTGATCTCT
chi-let-7g	100560	42530	22	TGAGGTAGTAGTTTGTACAGTT
chi-miR-499	88088	30477	21	TTAAGACTTGCAGTGATGTTT
chi-let-7i	79377	8212	22	TGAGGTAGTAGTTTGTGCTGTT
chi-let-7d	77925	12377	22	AGAGGTAGTAGGTTGCATAGTT
chi-miR-103	77519	15021	23	AGCAGCATTGTACAGGGCTATGA
chi-miR-495	75454	727	22	AAACAAACATGGTGCACTTCTT
chi-miR-107	71806	12072	23	AGCAGCATTGTACAGGGCTATCA
chi-miR-140	67024	49671	23	TACCACAGGGTAGAACCACGGAC
chi-miR-128	64515	17542	21	TCACAGTGAACCGGTCTCTTT
chi-miR-543	62573	489	22	AAACATTCGCGGTGCACTTCTT

Family analysis showed that the 464 known miRNAs belonged to 270 miRNA families in the two libraries. Most of the identified miRNA families have been shown to be conserved in a variety of species. For example, mir-9, mir-34, mir-124, mir-29 and let-7 families have been found in 72, 61, 73, 56, 68 species, respectively. However, some miRNAs have been shown to less conserved, such as miRNA-1246, miRNA-1260a, miRNA-1248, miRNA-1343 and miRNA-1307 only been found in 3, 2, 5, 5, 6 species, respectively (Table S3). In the present study, the largest miRNA family size identified was let-7 and miRNA-376, which consisted of 9 and 8 members, respectively. miRNA-2284, miRNA-2285 and miRNA-30 possessed 7, 6 and 7 members, respectively. The other miRNA families, such as miRNA-1, miRNA-221 and miRNA-206, had only one member.

**Figure 4:**
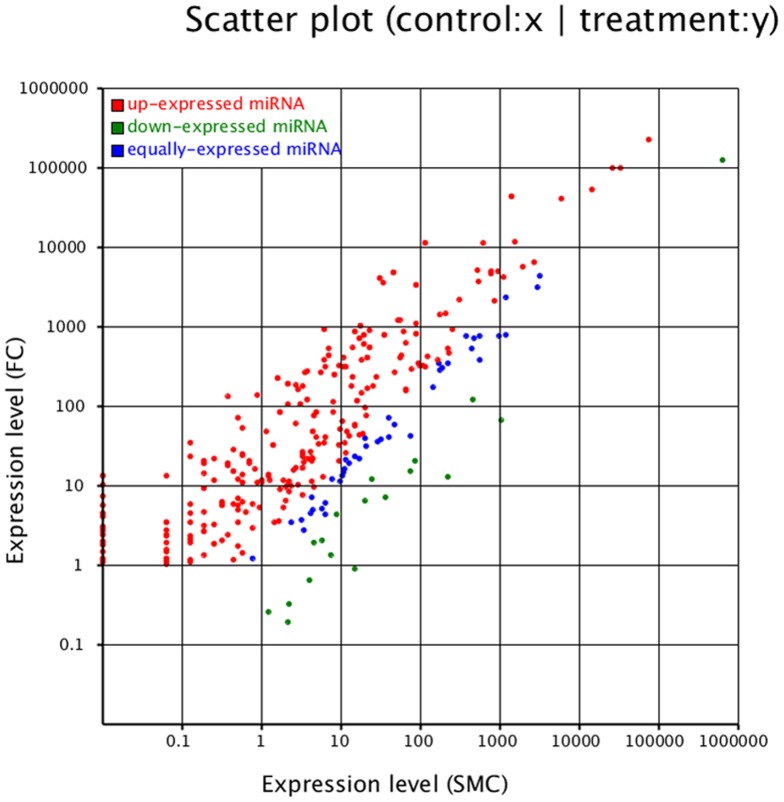
The differential expression of caprine known miRNA between fetal and six month old caprine muscle tissues. Expression level (SMC): Expression level of six month-old caprine musscle tissue; Expression level (FC): Expression level of fetal caprine muscle tissue. Red points represent miRNAs with a fold change>2, blue points represent miRNAs with 1/2<fold change≤2, green points represent miRNA with a fold change≤1/2.

To gain insight into possible stage-dependent roles of miRNAs during the development, we also investigated the differential expression profiles of the goat muscle tissue miRNAs. Expression of known miRNAs in the two libraries was demonstrated by plotting Log_2_-ratio and scatter plot (Figure 4). The expression profiles between the two libraries are shown in Table S4. According to the changes in relative miRNA abundance between the two libraries, 336 miRNAs are significantly differently expressed between FC and SMC (Table S4). Compared with miRNA expression in FC library, 317 miRNAs in SMC library were significantly down-regulated (Table S4). 26 miRNAs were significantly up-regulated in SMC library (Table S4). Among the up-regulated miRNAs, miR-487a has the highest fold-change (10 fold), while miR-29c has the highest fold-change (4 fold) in the down-regulated miRNAs.

### Identification and profiling of novel miRNAs candidates

The characteristic hairpin structure of miRNA precursors have been used to predict novel miRNAs. We predicted novel miRNAs by exploring the secondary structure, the Dicer cleavage site and the minimum free energy of the unannotated small RNA reads. A total of 4,447,299 unannotated sequences in the two libraries were used to predict novel miRNAs. We identified 83 potential novel miRNAs, which corresponded to 95 genomic loci. The length of the novel miRNA sequences ranged from 19 to 24 nt, with a distribution peak at 22 nt (Table S5). Among the 83 candidates, 74 novel miRNAs were identified in the fetal goat library and 36 were identified in the six month-old library, 27 miRNAs were expressed in both the two libraries (Table S5). 40 miRNAs were differentially expressed between the two development stages (Table S6). Compared with miRNAs expression in SMC library, 33 miRNAs were up-regulated in FC library, only 7 miRNAs were down-regulated.

The predicted hairpin structures of these novel miRNAs ranged from 65 to 99 nt in length and the folding energies ranged from −64.4 to −23.3 kcal/mol (Table S5). The read number for each novel miRNA was lower than that for the majority of known miRNAs. For example, the total reads of chi-novel-miRNA-30, chi-novel-miRNA-84 and chi-novel-miRNA-70 that had the highest reads number, were only 18540, 3436 and 3436, respectively. Moreover, the MFE index (MFEI) can be used for assaying miRNAs and distinguishing miRNAs from other coding and non-coding RNAs. We calculated the MFEI value for each individual miRNA precursor sequence. The values of MFEI for majority of miRNA precursors were greater than 0.85, a value that serves as a key discriminator to distinguish pre-miRNAs from other small RNAs.

### mRNA transcriptome profiling

miRNA regulates mRNA stability and translation. To further analyze the role of caprine miRNAs during muscle development, we characterized the mRNAs expression to do a miRNA-mRNA integrated analysis. After deep sequencing, we obtained 27,512,850 clean reads from the FC library and 27,582,908 clean reads from the SMC library (Table S7). Clean reads were mapped to a reference sequences using SOAPaligner/SOAP2. The ratio of reads mapped to reference genome was 67.92% for FC and 74.52% for SMC. 12,493,828 and 13,985,770 reads perfectly match the reference genome in the FC library and the SMC library (Table S7), respectively. 62.41% were unique match reads in the FC library as well as 66.89% in the SMC library. When mapped to the reference gene, the ratio of reads mapped to the reference gene was 68.18% for FC and 51.91% for SMC. 13,979,498 and 10,902,671 reads perfectly match the reference gene in the FC library and in the SMC library, respectively. 64.77% and 50.76% of mapped reads are unique matched reads in the FC library and SMC library (Table S7), respectively.

To reveal the molecular events during different development stages, genes that differentially expressed between the two libraries were identified. There are 7322 mRNAs with at least a two-fold difference in expression between FC and SMC libraries, of which 1329 genes are up-regulated, 5993 are down-regulated in the six month old caprine muscle tissue compared to that of the fetal, and 6907 are expressed in both libraries. The details of the gene-expression, including gene length, RPKM, log_2_ Ratio, *P* value and FDR were shown in Table S8.

### miRNA-mRNA integrated analysis

We firstly predicted the potential target genes of all the known miRNAs. Larger numbers of annotated mRNA transcripts were selected as potential targets for each library. Then, we screened out the potential targets of differentially expressed miRNAs which have a −3≥log2 ratio≥3. Then, these target genes were compared to the DEGs (listed in Table S8), and selected the intersection of the two gene sets and called them DE targets. The regulative relationship between these differentially expressed miRNAs and the DE targets were shown in Table S9. The result showed that most individual miRNAs had multiple gene targets and each target was regulated by multiple miRNAs. All DE-targets were then processed by KEGG pathway analysis. In total, 2719 targets significantly matched in the database, and were assigned to 231 KEGG pathways (Table S10). The pathways were listed according to the *P*-value in the table. It was shown that these DE-targets have a wide range of diverse functions.

**Figure 5:**
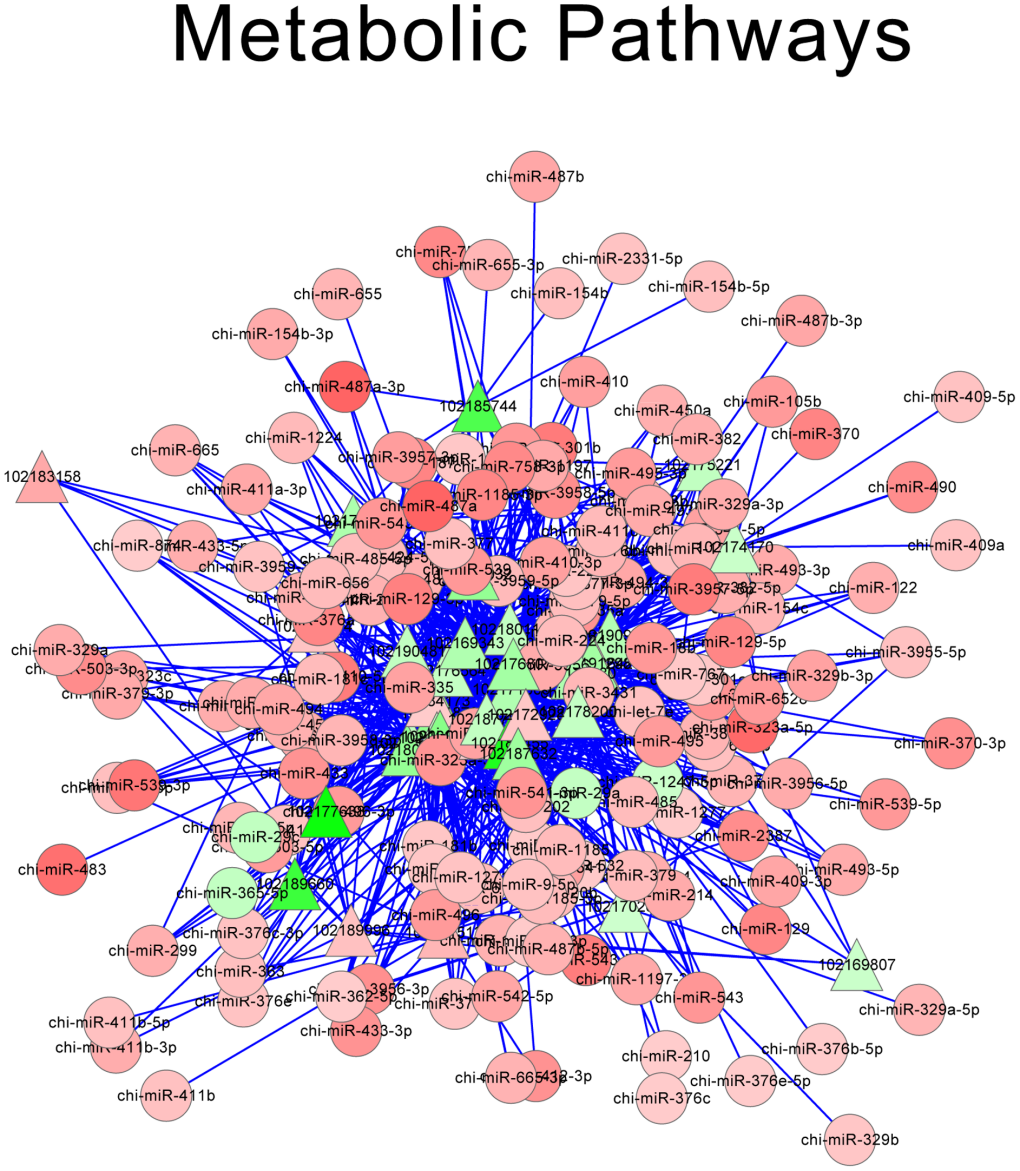
Network of metabolic pathways. Red triangle: up-regulated DE-targets; Green triangle: down-regulated DE-targets; Red roundness: up-regulated miRNAs; Green roundness: down-regulated miRNAs. The deeper the color, the stronger the trend.

**Figure 6:**
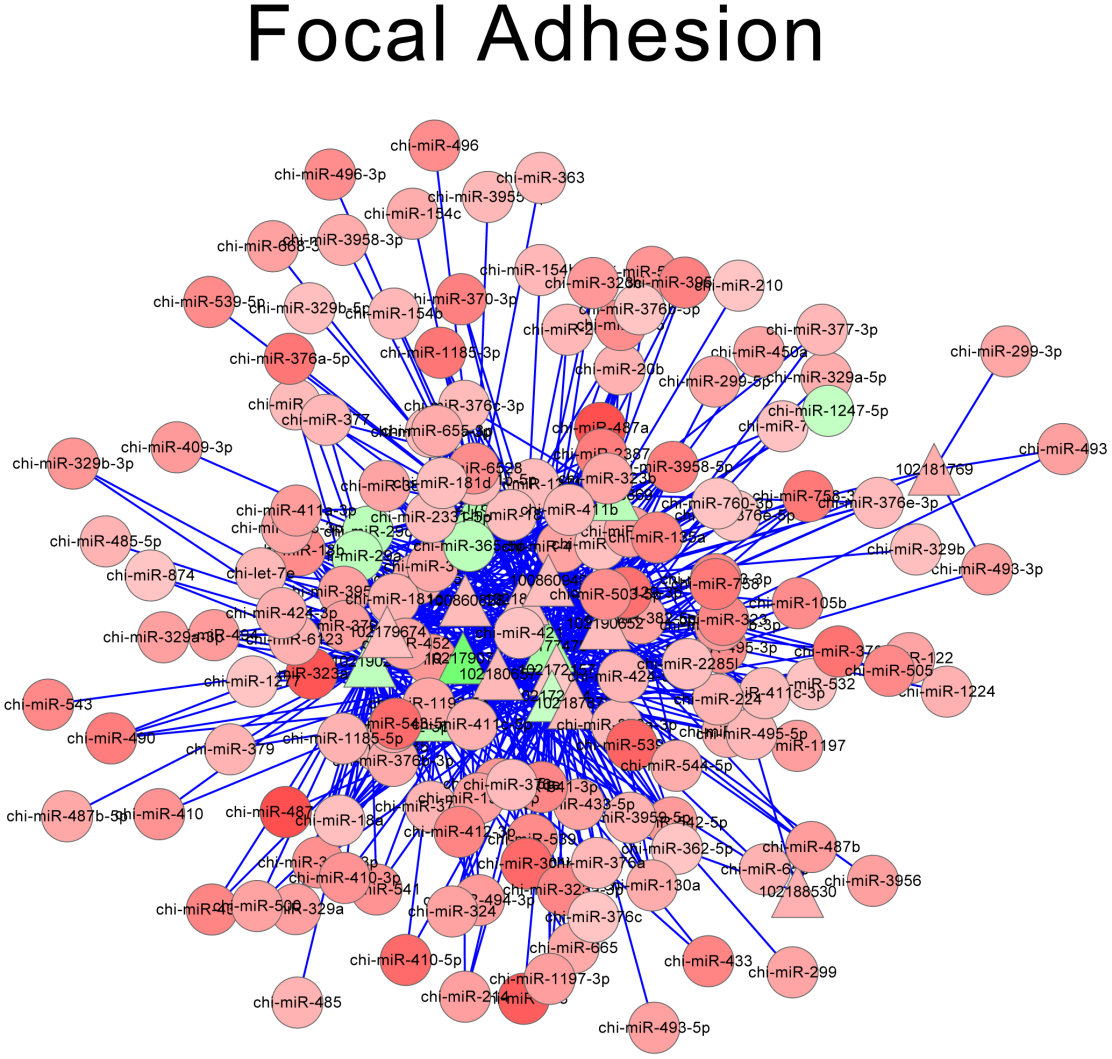
Network of focal adhesion. Red triangle: up-regulated DE-targets; Green triangle: down-regulated DE-targets; Red roundness: up-regulated miRNAs; Green roundness: down-regulated miRNAs. The deeper the color, the stronger the trend.

**Figure 7:**
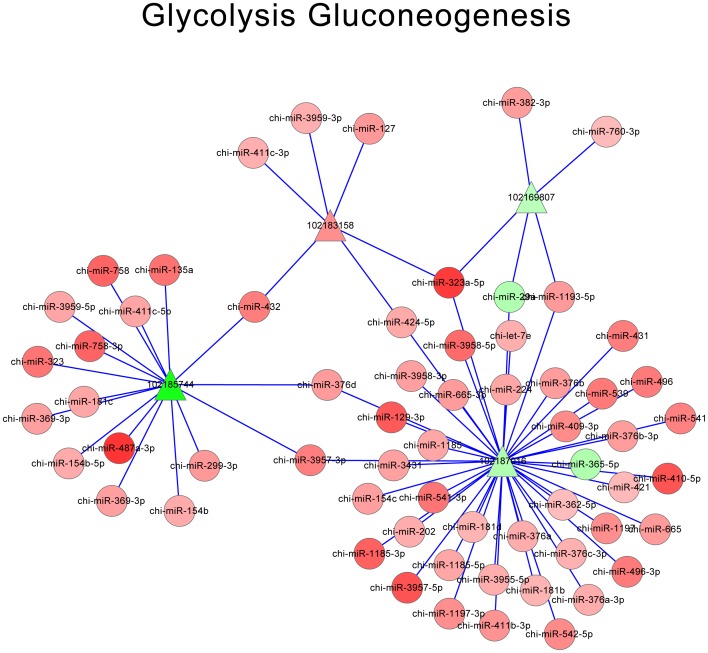
Network of glycolysis gluconeogenesis. Red triangle: up-regulated DE-targets; Green triangle: down-regulated DE-targets; Red roundness: up-regulated miRNAs; Green roundness: down-regulated miRNAs. The deeper the color, the stronger the trend.

**Figure 8:**
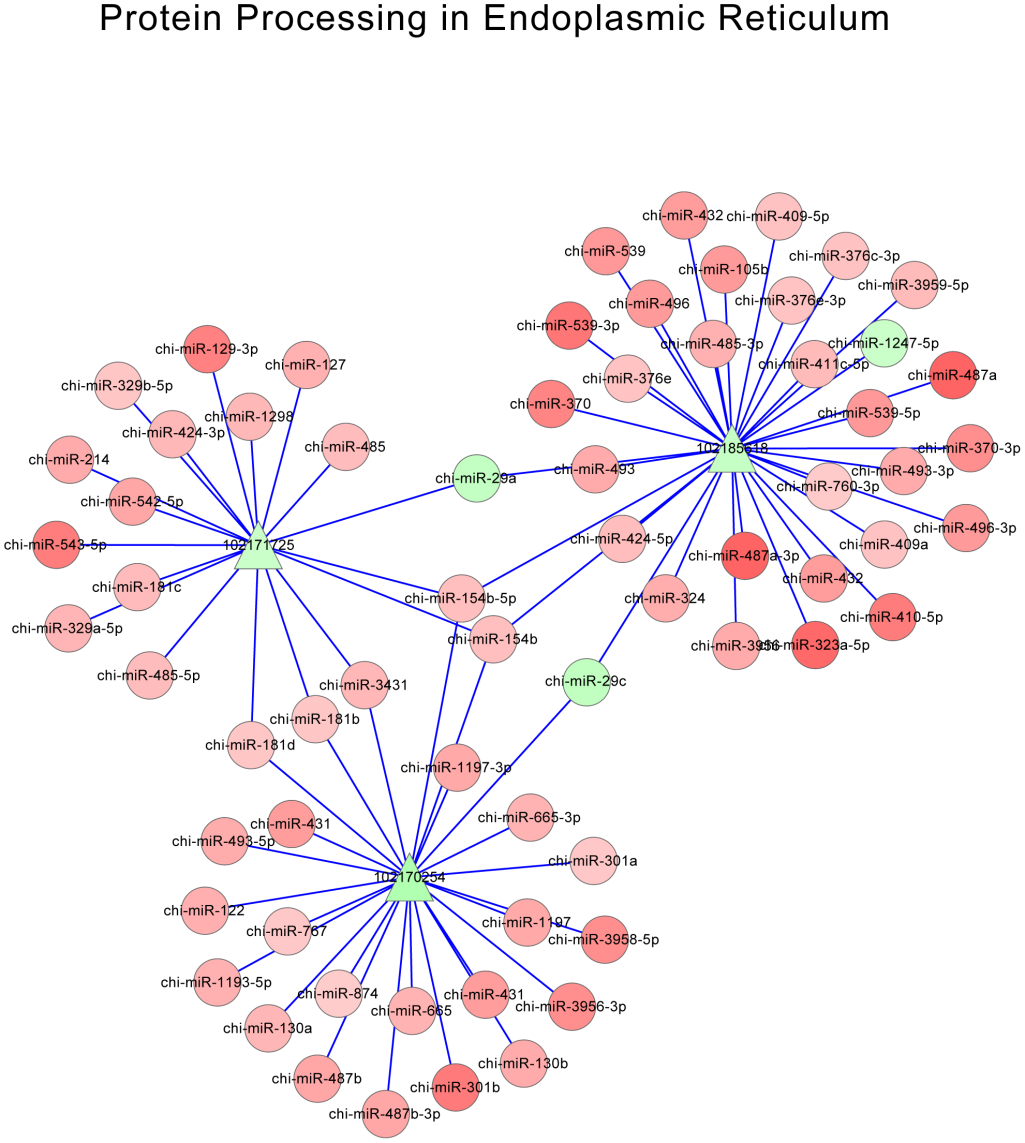
Network of protein processing in endoplasmic reticulum. Red triangle: up-regulated DE-targets; Green triangle: down-regulated DE-targets; Red roundness: up-regulated miRNAs; Green roundness: down-regulated miRNAs. The deeper the color, the stronger the trend.

According to miRNA-mRNA-KEGG annotation, 10 pathways with the smallest *P*-value were screened out, which were shown in Table 5. Each pathway comprised of multiple miRNAs and DE-targets. Then the interactions of miRNAs and DE-targets for these 10 pathways were integrated to construct possible regulatory networks, which were shown as network pictures. Net work of metabolic pathways, focal adhesion, glycolysis gluconeogenesis and protein processing in endoplasmic reticulum were shown in Figure 5, 6, 7 and 8, respectively. Oxidative phosphorylation, pentose phosphate pathway, ribosome, alzheimers disease, huntingtons disease, parkinsons disease were shown in Figure S1. The network of metabolic pathways included 169 miRNAs and 35 DE-targets (Table 5). In this pathway, miR-424-5p regulated most numbers (16) of DE targets, which suggested its important role in this pathway. When it came to focal adhesion (Figure 6), 163 miRNAs and 17 genes were involved in, and miR-424-5p regulated 14 DE-targets, which was the largest number of DE targets. It was very interesting that among the 7 networks, miR-424-5p were very active in that it regulates largest numbers of DE-targets and at the center of the networks. We also find miRNA-29a is involved in most of the networks. Except miR-424-5p and miRNA-29a, miR-129-3p, miR-181b and miR-181d were also involved in multiple pathways. This indicated that these miRNA might have very important biological function on myoblast proliferation or differentiation.

**Table 5:** Network of ten smallest *P*-value pathways.

Pathways	miRNA number	Targets genes number involved in network	*P*-value
Metabolic_pathways	169	35	3.25E-13
Focal adhesion	163	17	3.50E-07
Ribosome	38	5	8.44E-14
Oxidative phosphorylation	21	1	5.74E-08
Glycolysis Gluconeogenesis	65	4	2.51E-06
Pentose phosphate pathway	66	3	6.72E-06
Protein processing in endoplasmic reticulum	68	3	9.39E-06
Parkinson's disease	56	4	1.53E-11
Alzheimer's disease	54	3	1.53E-09
Huntington's disease	66	5	3.82E-09

### Validate the sequence data

#### Stem-loop RT-PCR for selected miRNAs

**Figure 9:**
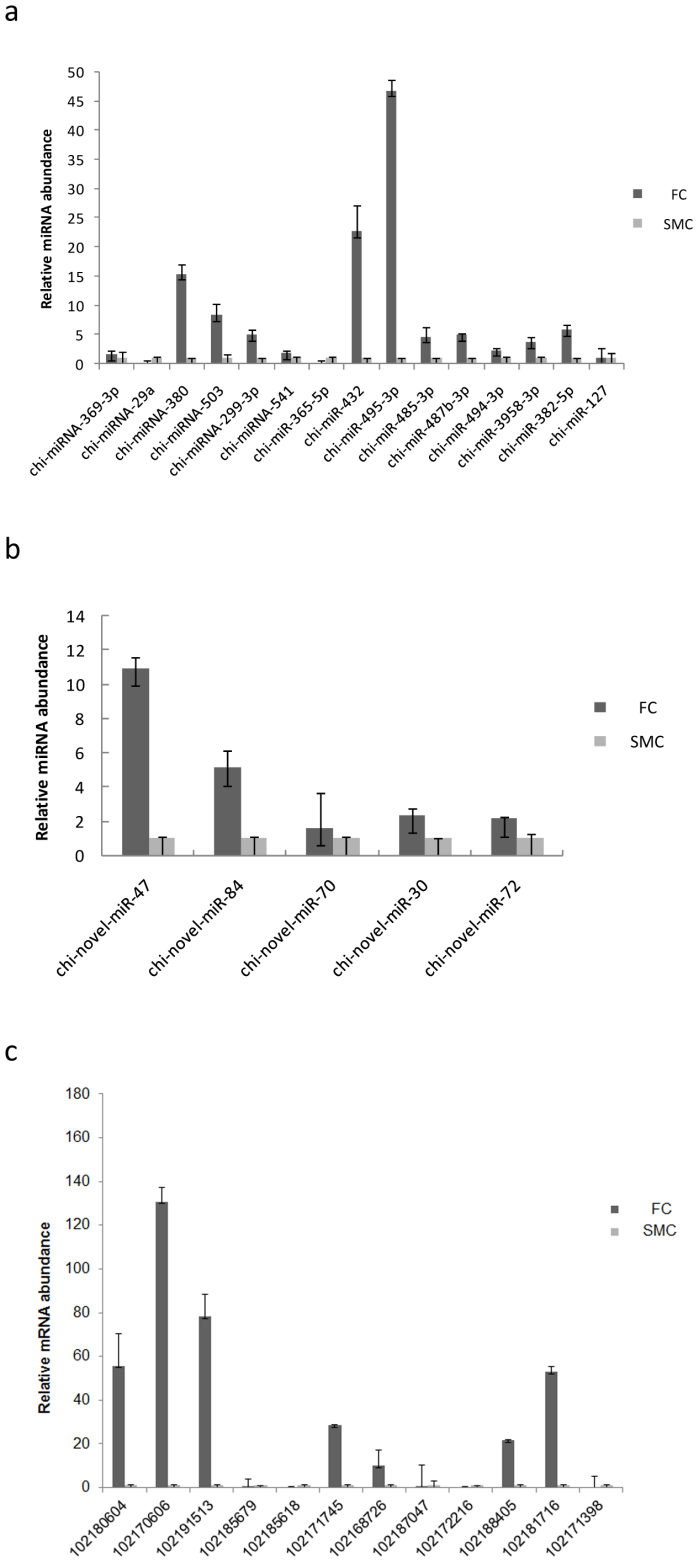
The expression of miRNAs and mRNAs in caprine muscle tissue were validated by RT-PCR. FC: fetal caprine; SMC: six month old caprine.

Stem-loop Real-time RT-PCR was performed on 15 differential expressed known miRNAs including chi-miR-485, chi-miR-487b, chi-miR-494, chi-miR-382-5p, chi-miR-432, chi-miR-495, chi-miR-365-5p, chi-miR-369-3p, chi-miR-29a, chi-miR-380, chi-miR-503, chi-miR-541, chi-miR-127, chi-miR-299-3p, chi-miR-3958, and 5 novel miRNAs, including chi-novel-miR-47, chi-novel-miR-84, chi-novel-miR-70, chi-novel-miR-30, chi-novel-miR-72. MiRNAs were chosen randomly. 18SrRNA was used as the internal reference RNA as it shows negligible expression differences along the sequence experiment. The sequences of primers were listed in the Table S11. As shown in Figure 9a, for the known miRNAs, the relative expression abundant of chi-miR-485, chi-miR-487b, chi-miR-494, chi-miR-382-5p, chi-miR-432, chi-miR-495, chi-miR-369-3p, chi-miR-380, chi-miR-503, chi-miR-541, chi-miR-127, chi-miR-299-3p and chi-miR-3958 were significantly higher in FC library than that of SMC library except chi-miR-29a and chi-miR-365-5p, which were highly correlated with sequence data. In addition, five novel miRNAs were chosen to detect the relative abundance. All of the five chosen miRNAs were shown to have higher expression level in FC library than in SMC library (Figure 9b).

#### Quantitative real-time PCR for selected DEGs

To measure gene expression levels, 12 genes were chosen to validate the sequence data using quantitative real-time PCR (Figure 9c). GAPDH gene was used as the internal reference gene as it shows negligible expression differences along the sequence experiment. The primer sequences are listed in Table S12. The result showed a highly correlation between sequence data and qPCR data (Figure 9c).

## Discussion

The development of the muscle results from the increase of muscle fibers and the expansion of cell volume, which entirely depend on the muscle cell proliferation and differentiation. Great deals of miRNAs have been reported to be enriched in muscle tissues and play an important role in the regulation of myoblast proliferation and differentiation. In recent years, the genomics of growth and development has gradually been elucidated through gene structure and function analyses to determine the gene expression regulation mechanism. miRNAs play critical roles in various biological and metabolic processes, by regulating gene expression at the post-transcriptional level. To date, many miRNA expression profiles have been reported in sheep, cow, chicken and pig, and other domestic animals in various tissues [43]–[46], but there is limited information about goat muscle miRNAs. The fetal stage is crucial for skeletal muscle development [47]. In livestock, all muscle fibers are formed during the prenatal stage, and 90-day around fetal life is the crucial stage of caprine myogenesis [48]. In contrast, postnatal skeletal muscle development is mainly due to the increase in muscle fiber size [49], and the period between birth and about eight months is the important stage of muscle fiber hypertrophy. Hence, in this study, 90 day fetal and six month old Huanghuai goat longissimus thoracis were collected for Solexa SBS technology sequencing.

The developmental skeletal muscle miRNA profile of several meat producing animal, including broiler, duck, pig, cattle and sheep, have been reported [28], [43], [50]–[52]. In the present study, 457 and 384 known miRNAs were detected in the library generated from fetal goat muscle tissue and six month old goat muscle tissue, respectively. Most of the sequences are 22nt in length, and majority of the small RNAs are 21–23nt in length, which is the typical size range of Dicer-derived products. The results are consistent with the small RNA distribution of Boer goat [53], sheep [54] and cattle [52], [55]. The most abundant miRNAs identified in our study were highly consisted with study on Boer goat, which reported that miR-133, miR-1, miR-378 and miR-206 families were the top 20 miRNAs in 6 month old Boer goat longissimus tissue [53], indicating the importance of these miRNAs on skeletal muscle development and growth. Interestingly, the most abundant miRNAs in our libraries were also highly consisted with that in bovine muscle libraries [52]. For instance, miR-1, miR-206, let-7f, let-7a-5p, let-7b, let-7c, let-7g, miR-378 etc, indicating that miRNA was highly conserved between cattle and goat. Thus, bovine *miRbase* could act as a credible reference for caprine miRNAs. The muscle specific miR-1, miR-133 and miR-206 are three of the most studied miRNAs up to now. Several studies demonstrate that these miRNAs are necessary for proper skeletal muscle development and function. MiR-1 or miR-206 promotes myogenic differentiation, while miR-133 over-expression enhances myoblast proliferation, but represses differentiation [10]–[11], [56]. Moreover, miR-1 and miR-133 have been implicated in skeletal muscle hypertrophy [14]. In the present study, the expression pattern of miR-206 and miR-133a are consistent with previous study on fetal bovine and adult bovine muscle tissue [52]. In addition, the expression level of miR-1 is significant higher (log2 ratio:−2.37) in six month old goat muscle. As six month-old is the important phase of muscle hypertrophy, we suppose that miR-1 may play an important role in caprine skeletal muscle hypertrophy. Moreover, many studies have reported that let-7 gene family is highly expressed and conserved across animal species, including mammals, flies, worms and plants [52], [57]–[59]. In the present study, let-7 gene family is sequenced at a high frequency in the caprine muscle tissue, these data are similar to those from other studies on miRNAs in different tissues, which indicated that the let-7 family is very important for some fundamental biological processes [58]. The family analysis of the identified miRNAs in our study showed that most members of conserved miRNAs families were expressed in caprine skeletal muscle, supporting the idea that regulatory or functional diversification has occurred [60]–[61].

Further analyzation of known miRNAs was conducted for nucleotide bias analysis. The base composition is an important feature of a nucleotide sequence for it can influence the physiochemical and biological properties of nucleotide sequences through effects on base pairing and related features, including thermodynamic folding of secondary structures. These characteristics, in turn, influence the biochemistry of nucleotide sequences as evidenced by the influence of secondary structure of miRNA [62]–[64]. In this study, characterization of the frequency of the position-specific nucleotide composition along the length of miRNA revealed that (Fig. 3) nucleotide positions 1 and 9 are significantly enriched for U relative to other bases, with a concordant depletion of G in FC library. Positions 2 to 8 of a mature miRNA is called the seed region, which is highly conserved. This “seed region” has been shown to be critical for miRNA targeting of mRNAs [65]. Positions 1 and 9 in mature miRNAs are adjacent both upstream and downstream of the “seed region”. Our results point to a potential role of these seed region flanking sequences in miRNA biogenesis or target site recognition. These were consisted with a previous report, who reported the dominance of U in position 1 and 9 [66]. This result showed that U was under strong selection at the ‘seed region’, and this selection may have important functions on either miRNA biogenesis or mRNA target recognition.

The known miRNAs were used for studying the expression profiles between the two development stages. A total of 336 miRNAs were differentially expressed between the two libraries, of which 317 were up-regulated and 26 were down-regulated in the FC library compared to the SMC library. Previous study about bovine muscle tissue reported 251 differentially expressed miRNAs consisted of 230 up expressed miRNAs and 21 down-expressed miRNAs in fetal bovine muscle tissue compared to adult bovine muscle tissue [52]. The result suggested that the miRNA concentration in the muscle tissue decreased in postnatal stage compared to the fetal stage. This is also consistent with previous studies that many miRNAs are highly expressed in the embryo as compared with that in adulthood, and the roles of these miRNAs are generally involved in embryo development and tissue identity maintenance [50], [67]–[68]. We speculated that the miRNAs differentially expressed between the FC and SMC may participate in regulating muscle tissue development and maintaining their distinct function. Thus, identifying the role and regulation of skeletal muscle miRNAs during various phases of muscle development, will significantly enhance our understanding of skeletal muscle biology and function.

In our database, 74 and 36 novel miRNAs were detected in the library generated from fetal goat and six month old goat muscle tissue, respectively. In addition, we found that a majority of pre-miRNA sequences had an MFE index (MFEI) values higher than 0.85, which were significant higher than other RNAs. This would be a better criterion for assaying and distinguishing miRNAs from other RNAs. Similar results in bovine have been reported [52]. Previous studies have shown that in plant and animals, the MFEI is a credible criterion for assaying miRNAs and distinguishing miRNAs from other coding and non-coding RNAs [52], [69].

The mature miRNAs exert their functions by binding the 3′UTR of the target mRNAs to degrade or repress their translation. Large numbers of miRNAs and their target gene may form a complex network, thus, the regulation between various miRNAs and mRNAs was complicated [70]. Establishing the interactions between miRNA and mRNA will enhance our understanding of embryonic and postnatal muscle development. In the present study, we employed a novel and generalizable method to efficiently identify functional miRNA-target interactions in caprine muscle tissue, namely, an integrated analysis of the miRNA profile and mRNA profile. To do the integrated analysis, we characterized the repertoire of differentially expressed transcripts in the six month old muscle tissue as compared to the fetal muscle tissue, and detected 7322 genes with at least a one-fold difference in expression between FC and SMC libraries (Table S8), which indicated that there were great differences between the two development stages. Several of the DEGs identified in our analyses were previously reported in the muscle tissue and some of them have been shown to affect muscle development [71]. For instance, the expression level of MRF and MEF2 families were significantly different between fetal stage and six month old stage. Through the integrated analysis, the interactions of differentially expressed miRNAs and target genes were shown in Table S9. We found that each miRNA had multiple targets, and each target was regulated by multiple miRNAs.

In order to further understand the miRNA-mRNA interactions, function of DE targets were determined. The Kyoto Encyclopedia of Genes and Genomes (KEGG) pathway database is a knowledge base for systematic analysis of gene functions in terms of networks of genes and molecules in the cells and their variants specific to particular organisms [72]. Genes within the same pathway usually cooperate with each other to exercise their biological function, which indicate that pathway-based analysis is a useful tool to further understand the biological functions of genes [73]. In our study, the top ten pathways that have the lowest *P*-value were mainly involved in the basic physiological and biochemical process of cell. The result also indicated that, except muscle specific miRNA (for instance, miR-1, miR-206, miR-133 etc), many non-muscle special miRNAs also played important roles in myocyte proliferation or differentiation through target some myogenic transcription factors, this was consist with previous studies that reported non-muscle-specific miRNAs regulate skeletal muscle differentiation [74], [75]. In the integrated analysis, the related miRNAs or DE-targets in each network were differentially-expressed miRNAs or genes, most of the miRNAs and DE-targets were involved in multiple pathways, for instance, miR-424-5p, miR-29a, bta-miR-129-3p, miR-181b and miR-181d. miR-424-5p was involved in most of the 10 pathways, and regulated the largest number of DE-targets. This indicated that miR-424-5p might play an important role in regulation muscle development. However, the biological characteristics, molecular mechanisms, and targets of miR-424-5p were not well-understood. miR-29a was a down-regulated miRNA and involved in most of the network. Previous study reported that miRNA-29a could suppress cell proliferation [76]. In brief, we described the network of miRNAs and DE-targets from a macroscopic view. miR-424-5p was involved in most of the pathways, and significantly differentially expressed during different development stages, suggesting its important regulatory effect. Moreover, except miR-424-5p and miR-29a, there were many other miRNAs that have important regulate effect in the network, further studies on these miRNAs would enable us to determine the mechanism of miRNAs in muscle development and miRNA-mRNA interaction.

## Conclusions

In conclusion, the data presented herein represent three advancements. First, using deep sequencing technologies, 464 known miRNAs were identified in skeletal muscle from fetal and six month old Huanghuai goat. The base composition analysis indicated that U is under strong selection in the position 1 and 9, which were the seed region flanking sequences, and this selection may have important function in either miRNA biogenesis or mRNA target recognition. Second, we have identified 83 novel miRNAs in longissimus thoracis from fetal and six month old goat. Most of the predicted novel miRNAs have an MEFI better than 0.85. Third, through intergrating the miRNA profile and the mRNA profile, the interaction of the miRNA and mRNA in this study were analyzed. Based on the networks, we found that miRNA-424-5p and miR-29a had important regulatory effect during muscle development.

### Data deposition details

The data set supporting the results of this article is available in the NCBI Gene Expression Omnibus (GEO, http://www.ncbi.nlm.nih.gov/geo/) repository, with access number GSE49258 (RNA-seq) and GSE49260 (small RNA-seq).

## Supporting Information

Figure S1 Network of miRNA and targets. Note: Red triangle: up-regulated DE-targets; Green triangle: down-regulated DE-targets; Red roundness: up-regulated miRNAs; Green roundness: down-regulated miRNAs. The deeper the color, the stronger the trend.
(PDF)

Table S1 Known miRNAs in this study.
(XLS)

Table S2 Nucleotide compositions of known miRNAs in this study.
(XLSX)

Table S3 Family analysis of known miRNA.
(XLSX)

Table S4 Known miRNAs expression profiles of the two libraries.
(XLS)

Table S5 Novel miRNAs identified in this study.
(XLSX)

Table S6 Novel miRNAs expression profiles.
(XLS)

Table S7 Statistics of alignment (map to reference genome and gene).
(DOCX)

Table S8 Differentially expressed genes that have FDR≤0.001 and Fold Change >2.
(XLS)

Table S9 MiRNA-mRNA integrated analysis.
(XLSX)

Table S10 Pathway of differential expression targets.
(XLSX)

Table S11 The primer sequences of the stem-loop qPCR experiments.
(XLS)

Table S12 The primer sequences of the qPCR experiments.
(XLS)
