# Supplementary material for: Identification and Profiling of microRNAs and Their Target Genes from Developing Caprine Skeletal Muscle
Source: PLoS One. 2014 May 12;9(5):e96857. doi: 10.1371/journal.pone.0096857 (PMC4018397; doi:10.1371/journal.pone.0096857)

Pentose Phosphate Pathway

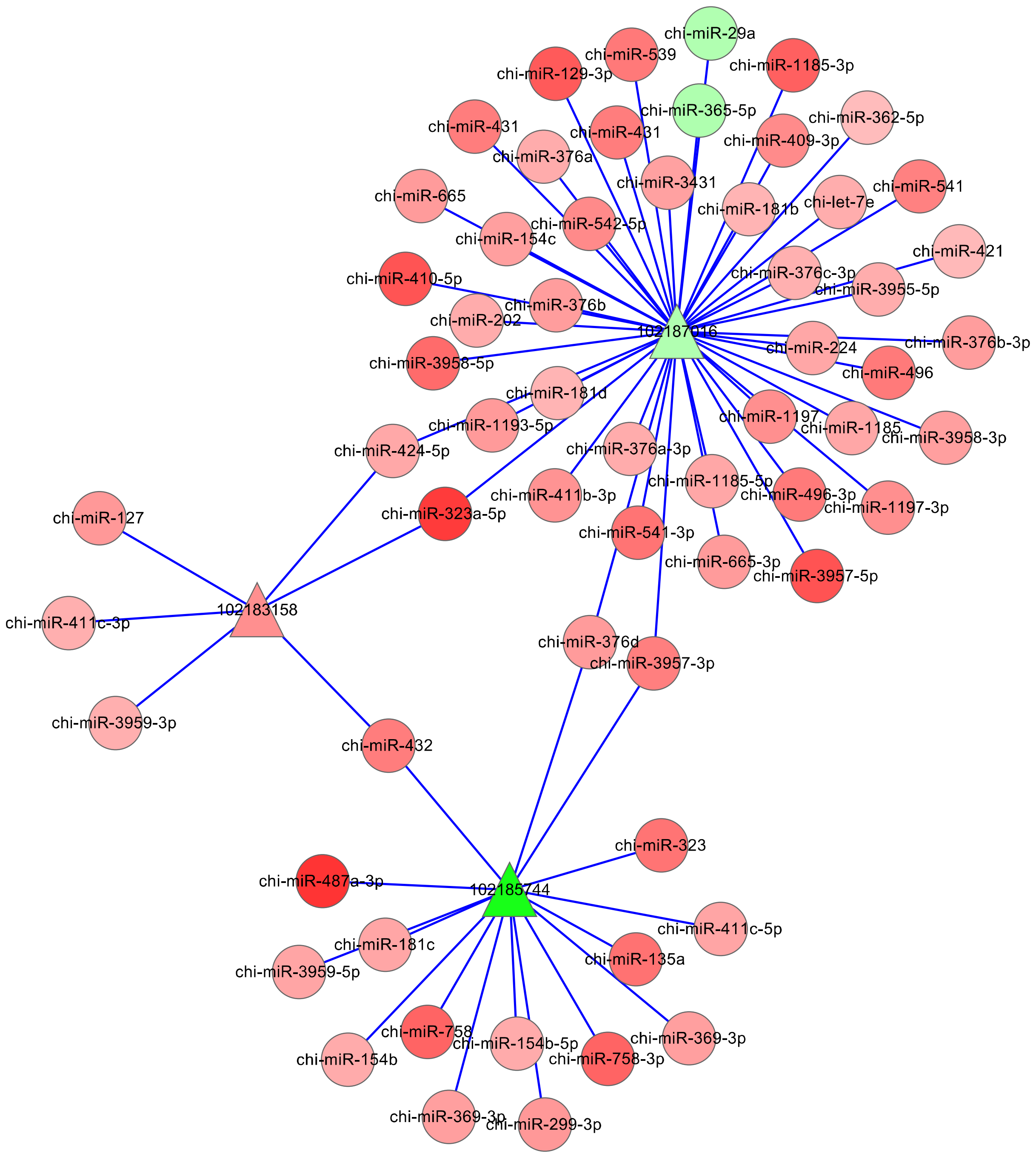

Alzheimers Disease

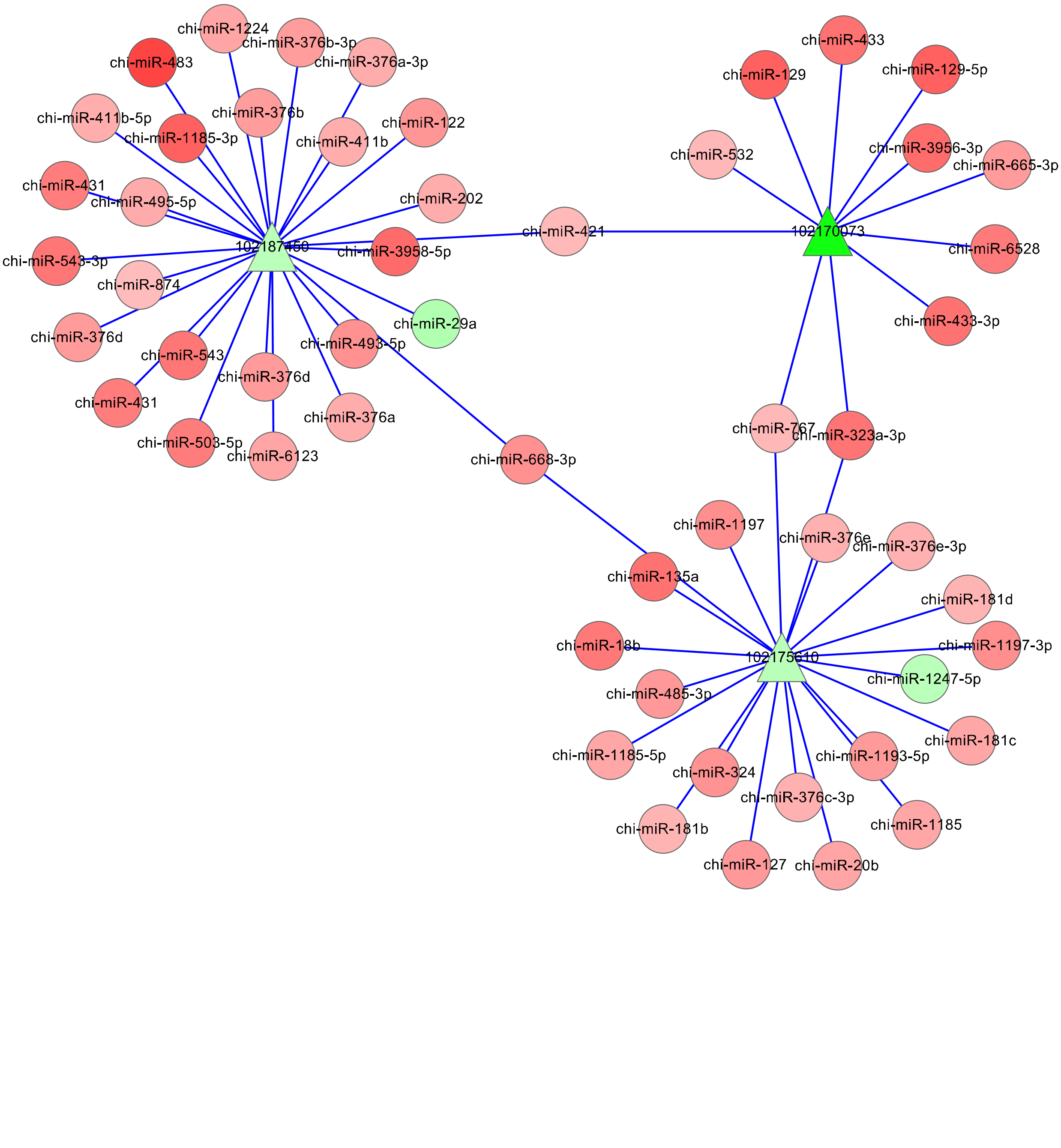

Huntingtons Disease

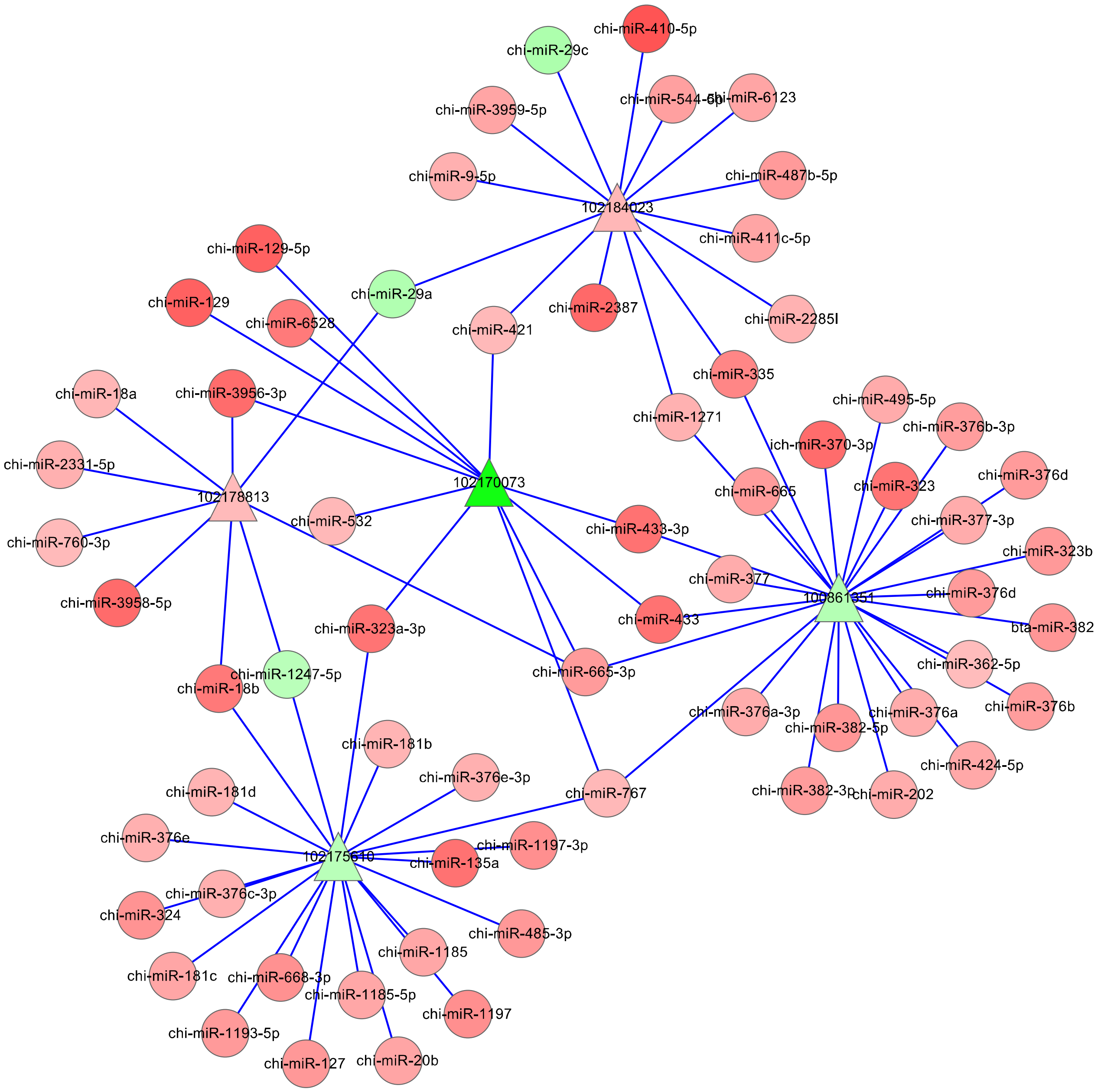

Parkinson's Disease

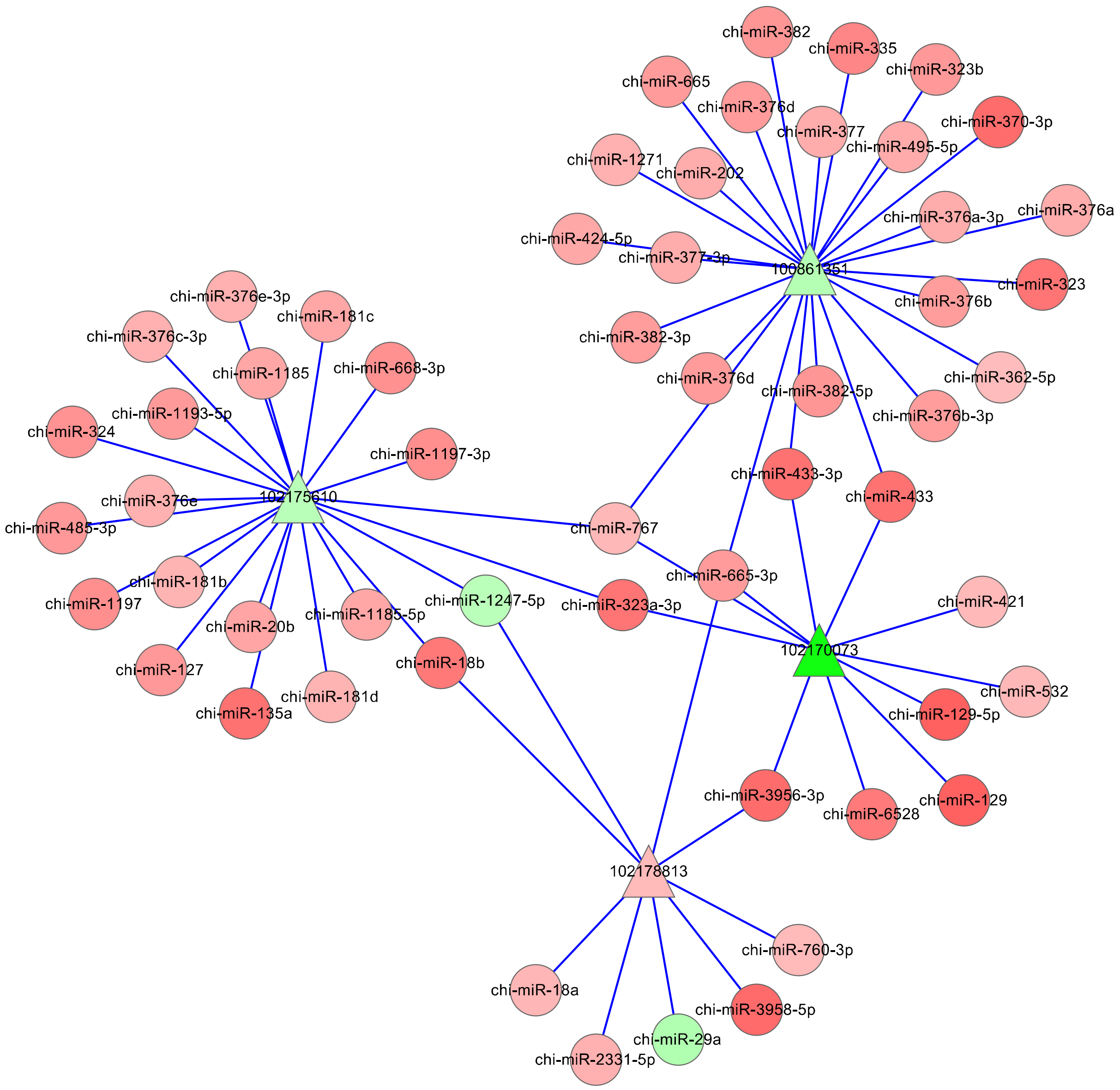

Ribosome

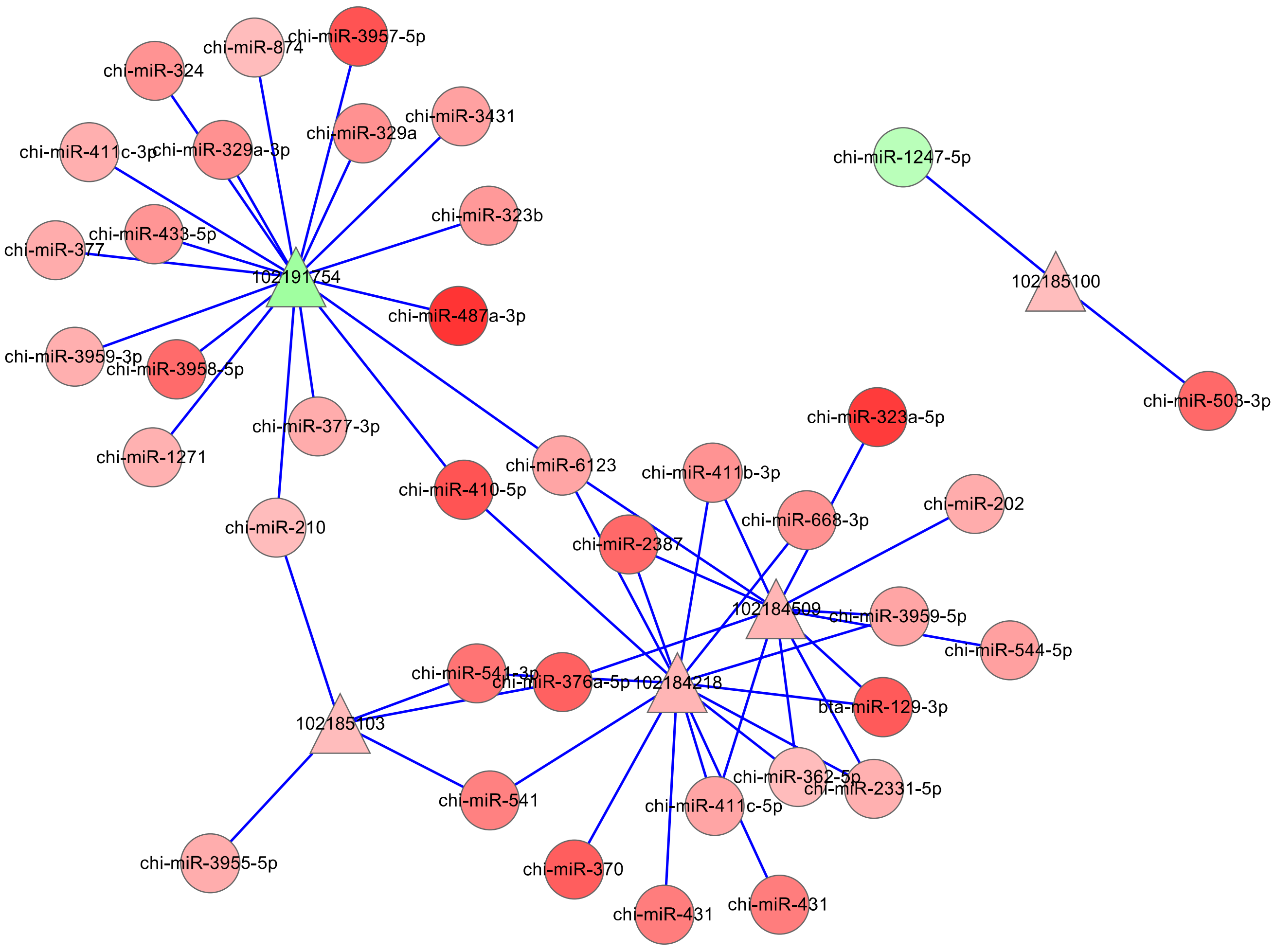

chi-miR-3957-5p
chi-miR-874
chi-miR-324
chi-miR-3431
chi-miR-329a
chi-miR-411c-3p
chi-miR-329a-3p
chi-miR-1247-5p
chi-miR-323b
chi-miR-433-5p
chi-miR-377
102191754
102185100
chi-miR-487a-3p
chi-miR-3959-3p
chi-miR-3958-5p
chi-miR-503-3p
chi-miR-323a-5p
chi-miR-377-3p
chi-miR-1271
chi-miR-6123
chi-miR-411b-3p
chi-miR-410-5p
chi-miR-668-3p
chi-miR-202
chi-miR-210
chi-miR-2387
102184509
chi-miR-3959-5p
chi-miR-544-5p
chi-miR-541-3p
chi-miR-376a-5p
102184218
bta-miR-129-3p
102185103
chi-miR-362-5p
chi-miR-2331-5p
chi-miR-541
chi-miR-411c-5p
chi-miR-3955-5p
chi-miR-370
chi-miR-431
chi-miR-431

Oxidative Phosphorylation

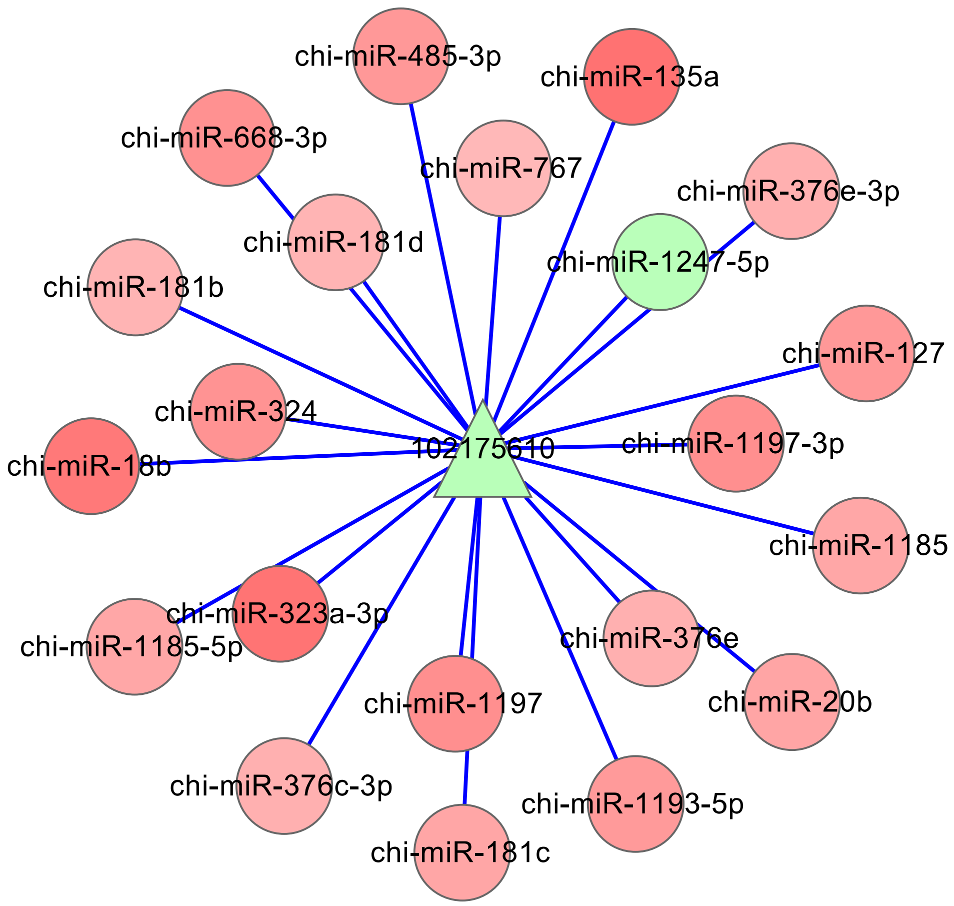

Supplement: Figure S1 — Network of miRNA and targets. Note: Red triangle: up-regulated DE-targets; Green triangle: down-regulated DE-targets; Red roundness: up-regulated miRNAs; Green roundness: down-regulated miRNAs. The deeper the color, the stronger the trend. (PDF) [file pone.0096857.s001.pdf]
