# Supplementary material for: Identification and Profiling of microRNAs and Their Target Genes from Developing Caprine Skeletal Muscle
Source: PLoS One. 2014 May 12;9(5):e96857. doi: 10.1371/journal.pone.0096857 (PMC4018397; doi:10.1371/journal.pone.0096857)
Supplement: Table S7 — Statistics of alignment (map to reference genome and gene). (DOCX) [file pone.0096857.s008.docx]

Table S7 Statistics of alignment (map to reference genome and gene)

**Statistics of alignment (map to reference genome)**

| **Map to Genome** | **Fetal caprine muscle tissue** | | **Six month caprine muscle tissue** | |
| --- | --- | --- | --- | --- |
|  | **reads number** | **percentage** | **reads number** | **percentage** |
| Total Reads | 27512850 | 100.00% | 27582908 | 100.00% |
| Total BasePairs | 2476156500 | 100.00% | 2482461720 | 100.00% |
| Total Mapped Reads | 18686741 | 67.92% | 20553944 | 74.52% |
| perfect match | 12493828 | 45.41% | 13985770 | 50.70% |
| <=3bp mismatch | 6192913 | 22.51% | 6568174 | 23.81% |
| unique match | 17170837 | 62.41% | 18450311 | 66.89% |
| multi-position match | 1515904 | 5.51% | 2103633 | 7.63% |
| Total Unmapped Reads | 8826109 | 32.08% | 7028964 | 25.48% |

**Statistics of alignment (map to reference gene)**

| **Map to Gene** | **Fetal caprine muscle tissue** | | **Six month caprine muscle tissue** | |
| --- | --- | --- | --- | --- |
|  | **reads number** | **percentage** | **reads number** | **percentage** |
| Total Reads | 27512850 | 100.00% | 27582908 | 100.00% |
| Total BasePairs | 2476156500 | 100.00% | 2482461720 | 100.00% |
| Total Mapped Reads | 18758520 | 68.18% | 14318233 | 51.91% |
| perfect match | 13979498 | 50.81% | 10902671 | 39.53% |
| <=5bp mismatch | 4779022 | 17.37% | 3415562 | 12.38% |
| unique match | 17820015 | 64.77% | 14001993 | 50.76% |
| multi-position match | 938505 | 3.41% | 316240 | 1.15% |
| Total Unmapped Reads | 8754330 | 31.82% | 13264675 | 48.09% |
